# The fuzzy brain. Vagueness and mapping connectivity of the human cerebral cortex

**DOI:** 10.3389/fnana.2012.00037

**Published:** 2012-09-05

**Authors:** Philipp Haueis

**Affiliations:** Max Planck Research Group “Neuroanatomy and Connectivity”, Max Planck Institute for Cognitive and Brain SciencesLeipzig, Germany

**Keywords:** connectivity, cytoarchitectonics, fuzzy boundaries, neuroanatomy, statistical thresholding, vagueness

## Abstract

While the past century of neuroscientific research has brought considerable progress in defining the boundaries of the human cerebral cortex, there are cases in which the demarcation of one area from another remains fuzzy. Despite the existence of clearly demarcated areas, examples of gradual transitions between areas are known since early cytoarchitectonic studies. Since multi-modal anatomical approaches and functional connectivity studies brought renewed attention to the topic, a better understanding of the theoretical and methodological implications of fuzzy boundaries in brain science can be conceptually useful. This article provides a preliminary conceptual framework to understand this problem by applying philosophical theories of vagueness to three levels of neuroanatomical research. For the first two levels (cytoarchitectonics and fMRI studies), vagueness will be distinguished from other forms of uncertainty, such as imprecise measurement or ambiguous causal sources of activation. The article proceeds to discuss the implications of these levels for the anatomical study of connectivity between cortical areas. There, vagueness gets imported into connectivity studies since the network structure is dependent on the parcellation scheme and thresholds have to be used to delineate functional boundaries. Functional connectivity may introduce an additional form of vagueness, as it is an organizational principle of the brain. The article concludes by discussing what steps are appropriate to define areal boundaries more precisely.

## Introduction

In a recent article on the advantages of using functional regions of interest (fROI) in human brain imaging, Rebecca Saxe, Mathew Brett, and Nancy Kanwisher caution the interested layman not to misuse or over-interpret neuroscientific results:

The “activation map” images that commonly accompany brain imaging papers can be misleading to inexperienced readers, by seeming to suggest that the boundaries between “activated” and “unactivated” patches of cortex are unambigous and sharp. Instead, as most researchers are aware, the apparent sharp boundaries are subject to the choice of threshold applied to the statistical tests that generate the image. What, then, justifies dividing the cortex into regions with boundaries based on this fuzzy, mutable measure of functional profile?

(Saxe et al., 2010, p. 39).

Saxe and colleagues proceed their discussion in pointing out that it is an empirical question whether fROIs have precise boundaries or not, and that neuroscientists do not have to avoid fuzziness, since it is “a feature of many scientifically respectable objects, including ocean currents like the Gulf Stream, geographical features like Mt. Fuji, and human body parts like knees and elbows” (Saxe et al., 2010). As long as the neuroscientific community reaches consensus about what they are referring to, fuzziness in the brain seems just a usual feature of scientific research.

The problem of imprecisely defined (scientific) objects is known in philosophy under the name of “vagueness”: concepts are vague when they allow borderline cases of application. For these cases it is unclear whether the concept still applies or not, independent from the lack of knowledge. Saxe and colleagues are correct in assuming that vagueness is a ubiquitous phenomenon in both scientific and everyday language (Russell, 1923). But in claiming that vagueness is merely an empirical issue which requires a consensual solution, and in not distinguishing vagueness from ambiguity or uncertainty due to technological shortcomings, they also express an opinion which is indicative of how most neuroscientists think about boundaries of cortical areas in general (and not just of fROIs). Since the theoretical literature on vagueness and its application to fields as different as law and geography (Endicott, 2001; Varzi, 2001) seems largely unknown to neuroscientists, this paper attempts to provide a preliminary conceptual framework to understand the phenomenon of vagueness in neuroanatomy (“The Philosophical Concept of Vagueness”).^1^ “Gradual Transitions in Cytoarchitectonic Studies of Brain Anatomy” and “Thresholds and Measurement of Neural Activity with fMRI” describe vagueness at two levels of neuroscientific research, namely for (1) cytoarchitectonics, where cortical areas are identified at the level of neurons and for (2) voxel activation at the level of intra-areal neuronal interaction measured by the blood oxygen level dependent (BOLD)-signal of functional magnetic resonance imaging (fMRI). “Implications of Vagueness for Mapping Connectivity of the Human Cerebral Cortex” proceeds to discuss the implications of (1) and (2) for distinguishing areas on the level of inter-areal connectivity. “Anatomical Connectivity and Overlapping Networks” explores vagueness in recent attempts to define networks of anatomical connectivity using structural MRI and diffusion tractography. “Functional Connectivity I: Edge Detection Algorithms and Functional Boundaries” discusses the use of resting state fMRI and computer vision algorithms to delineate functional boundaries between cortical areas. “Functional Connectivity II: Research Contexts and Combinatory Vagueness” discusses further the conceptual vagueness of the term “functional connectivity.” The paper concludes by arguing that when these implications of vagueness are reflected, this may help to understand which attempts of defining areal boundaries more precisely are admissible and methodologically sound.

## The philosophical concept of vagueness

In order to understand what kinds of vagueness arise at different levels of neuroanatomical research, some general distinctions from the philosophical literature need to be introduced. The classical form of degree-vagueness leads to the so-called “Sorites-paradox” (for an overview see Keefe and Smith, 1996): as often explicated based on the paradigmatic example of a heap (greek: *soros*), a clear case of applying a concept (e.g., “10,000 grains of sand make up a heap”) is taken as the premise of an argument which proceeds by small steps (“If 10,000 grains of sand are a heap, 9999 grains of sand are a heap too”) toward a paradoxical conclusion (“0 grains of sand are a heap”). At some point in this series the concept failed to apply, but exactly where its extension ended is impossible to tell because there are borderline cases (e.g., “7 grains of sand make up a heap”) for which it is indeterminate whether the propositions expressed by the sentences that contain vague predicates are true or false (they have so-called truth value gaps cf. Tye, 1990). The class of borderline cases itself shares a fuzzy boundary with the clear cases and non-cases, which is called “higher-order-vagueness” (Williamson, 1994). Distinct from the vagueness concerning the extension of predicates (i.e., the class of objects which fall under a certain concept) is the so-called “problem of the many” (Unger, 1980). Here it is unclear whether a certain material part belongs to an object (e.g., a chunk of rock at the base of Mt. Fuji). Because of their fuzzy spatiotemporal boundaries, it is impossible to unequivocally pick out these objects amongst others of their kind, which is why this form is called “vagueness of individuation” (or object vagueness). Common to both forms is that they (a) allow for borderline cases (of application/membership), (b) have fuzzy boundaries (extensional/spatiotemporal), and (c) that the objects/concepts in question are tolerant to small changes. Degree-vagueness can furthermore arise both from categorical properties, like the number of grains of sand, or gradable ones, like the shade of a color. The attempt to resolve degree-vagueness by introducing many dimensions can lead to combinatory vagueness, where it is unclear which or how many conditions have to be fulfilled to pick out an object or apply a concept (see section “Functional Connectivity II: Research Contexts and Combinatory Vagueness”). Additionally, predication and object vagueness can be understood to be semantic, i.e., a resulting from the imprecision of language (Russell, 1923) or to be ontic, i.e., resulting partly from the world itself, because it is such that knowledge about the presence or absence of a certain property is impossible (Hyde, 2007). Since the question of ontic and semantic vagueness is controversially discussed in the philosophical literature, the preliminary framework given here is as neutral as possible to which type is more appropriate to understand vagueness in neuroanatomy. It is more important for the present purposes that vagueness is to be distinguished from epistemic uncertainty, where indeterminacy merely arises through incomplete knowledge or technological shortcomings. In the empirical sciences, these are usually co-existing and overlapping types of indeterminacy.

## Gradual transitions in cytoarchitectonic studies of brain anatomy

When dealing with vagueness, it is always important to keep in mind that it does not imply that for every case it is uncertain whether to apply a certain concept (e.g., “heap” to 10,000 grains of sand), or whether a certain material part belongs to an object (e.g., the peak of Mt. Fuji). The same is true for the anatomy of the brain, where non-peripheral points can be easily identified as being part of an area in question, and where examples of well-defined and clearly delineated areas such as the primary visual cortex exist (Hinds et al., 2009). But besides these clear examples, the problem of gradual transitions between cortical areas has been known since the early cytoarchitectonic studies of brain structure, where histological sections of post-mortem brains were stained to reveal differences in the laminar pattern of different areas, based e.g., on size, shape, and density of neuronal cells, or relative/absolute thickness of the cortex. Korbinian Brodmann, the author of one of the most well-known and widely used parcellation schemes of the human cortex, took a decidedly pragmatic position on the issue of determining boundaries in the brain. Although noticing that the cytoarchtitectonic features mentioned above sometimes change abruptly and sometimes gradually between areas, he maintained that “nevertheless the maps bring the position and the mutual relations between the regions correctly into view, and everyone will be able to apply them with great utility in comparative studies, as long as one does not search more in it than they should be: *a tool for orientation*” (Brodmann, 1909, p. 126, emphasis in original, my translation). In the mid of the last century, von Bonin and Bailey (1951) drew the more radical conclusion that the great variability of cytoarchitectonic features prohibits to draw any sharp boundary in the human cortex, which was a common feature of most former brain atlases (Campbell, 1905; Smith, 1907; Economo and von Koskinas, 1925; Vogt and Vogt, 1925; Sarkissov et al., 1949). While abandoning any precise demarcations may have been indeed a too radical conclusion, the recognition of gradual transitions by these prominent historical figures indicates that the spatial distribution of cortico-anatomical properties can be crucial for a systematic understanding of vagueness in neuroanatomy.

Consider the following example: A characteristic feature of Brodmann's area 4 is the presence of giant pyramidal cells (Betz-cells) in layer V, but their size varies considerably between different individuals, both in height (60–120 μm) and width (30–60 μm) (Amunts et al., 2002). But even in a single individual, Betz-cells can be found outside of area 4, and the distance between them increases toward area 6 (Economo and von Koskinas, 1925; Zilles et al., 1995). If the concept of a cortical area is defined as a homogenous and architectonically distinct region in the brain (Amunts et al., 2002) then it becomes apparent why the definition of area 4 based on the Betz-cell criterion is multiply vague. First, height and width are gradable properties that introduce indeterminacy into the definition of what a Betz-cell is (equivalent to how big a grain of sand can be to still count as sand). Thus, a Sorites-series could be constructed ordering the slices from different individuals according to the size, in which there would be borderline cases of how big or small a Betz-cell can be at each end of the series. The size vagueness of Betz-cells itself does not pose an issue for delineating the cortical boundary of area 4, however, because it only arises by comparing different individuals, while for each single slice the Betz-cells can be precisely identified (see below). The more severe form of vagueness is that by following the increasing distance of Betz-cells toward area 6 in a single slice, area 4 cannot be precisely individuated: at the periphery there are parts such that it is indeterminate whether the proposition expressed by the sentence “X is part of area 4” is true or false. At the end of the movement from area 4 to 6 is a point for which it is clearly false to say it belongs to area 4, but which has been derived from following the presence of Betz-cells in a step-wise manner. Of course neuroanatomical research is in practice preserved from the paradoxical conclusion by considering additional cytoarchitectonic modalities such as granularity as well as myeloarchitecture (Sanides, 1964), receptor binding sites (Jansen et al., 1991), or integrating areas into mechanistic explanations by specifying their functional roles in cognitive tasks (Craver, 2007). But to discard the Sorites-paradox entirely because of these possibilities would be to miss the idea of the argument, because in the case of the heap, one property is picked out while others are being held constant or regarded as irrelevant (such as shape of the pile or color of the grains). It can be still maintained that the one-dimensional gradual transitions of a property along the cortex is the basis for the phenomenon of vagueness in neuroanatomy. Although multi-modal approaches often reduce the problem of gradual transitions to practically manageable issues, they could still be facing multidimensional vagueness (see section “Functional Connectivity II: Research Contexts and Combinatory Vagueness”), because it can be unclear which dimension should be prioritized when modalities point toward different boundary locations.

Gradual transitions of cytoarchitectonic properties become a challenge for brain mapping, however, when they are distributed in a way that a whole cortical area is considered a transitional zone because it shares some, but not all cytoarchitectonic properties of its adjacent neighbors. A prominent example is the transitional area 9/46 in the human prefrontal cortex (Rajkowska and Goldman-Rakic, 1995a,b). This area shares with area 9 a pale sublayer Vb, and with area 46 a distinct layer IV and uniformely sized cells in layers III and V. Additionally, this transitional area shares combined features at the periphery toward other areas (classified as 9–8, 9–45, 46–10, and 46–45) and cannot be separated distinctly to areas 9 or 46 based on myeloarchitecture.^2^ Thus, both of Brodmann's traditional designations represent cases of extensional vagueness where borderline cases have been observed already (in contrast to intensional vagueness, where borderline cases are merely possible, cf. Fine, 1975): it is indeterminate for parts now designated as area 9/46 whether they belong to area 9 or 46, respectively. But despite the persistence of these cases in light of additional anatomical modalities, the extensionally vague boundary between area 9 and 46 remains relative, since until now there has been no parcellation of these areas based on the differential distribution of receptor binding sites. There are good reasons, however, to regard this case as an absolute case of vagueness—where evidence from all modalities reveals an indeterminacy—although this is an hypothesis which has to await further empirical research. First, albeit chemoarchitecture can show abrupt transitions between well-defined areas (such as V1 and V2, Zilles et al., 2002), not all receptors show areal boundaries equally (Zilles et al., 1995), and some are also heterogeneously distributed within an area (e.g., GABA_a_ receptors within area V1, Zilles and Schleicher, 1993). Second, even if these receptor bindings reveal differences within this transitional area, functional segregation alone does not constitute an architectonic entity (Amunts et al., 2002), partly because mechanistic explanations of functions usually do not consider organizational features that are irrelevant to the explanandum phenomenon (cf. Craver, 2007, p. 144). Third, according to Rakic, the transitional area 9/46 may also represent a newly evolving structure in the brain, which therefore does not yet reveal any distinguishing features (Rakic, 1988). Considering these constraints to precisely determine which parts of 9/46 belong decisively to one of the neighboring areas, it seems reasonable to assume that, for any meaningful timescale of actual research, the architectonic definitions of areas 9 and 46 based on structural cell properties will remain vague.^3^ Additionally, the introduction of a transitional zone only collects all the borderline cases from the neighboring areas (which is itself a vague class, see section “The Philosophical Concept of Vagueness”), instead of providing a solution of how to deal with a fuzzy boundary in each case.

In summary, it becomes evident that gradual transitions are a well-known issue in the cytoarchitectonic study of the brain, and that they can be illuminated by applying the conceptual framework of the philosophical vagueness literature. Notice that vagueness in these cases is closely related, but not identical to the uncertainty of measurement arising from inter-individual variability. It is certainly one of the biggest challenges of neuroanatomy to describe higher cortical areas, such as Broca's region (Brodmann area 44 and 45), whose position and size varies highly, additionally to subtle changes of cell properties, between different individuals (Fischl et al., 2008). Although this variability makes locational assignments in functional imaging studies problematic (Devlin and Poldrack, 2007), the vagueness here described does not arise from comparative studies, because the cytoarchitectonic criteria fail to identify an area's position precisely even in the case of a singular instance (Schneider and Erwig, 1997). Probabilistic cytoarchitectonic maps do not address the issue of gradual transitions either, because they do not deal with imprecision on the individual but on the group level, which is why they face the problem of arbitrary thresholding (see section “Gradual Transitions in Cytoarchitectonic Studies of Brain Anatomy”).

## Thresholds and measurement of neural activity with fMRI

The cautionary remark of Saxe and colleagues quoted in the introduction was concerned with the allegedly sharp and unambiguous localization of cognitive functions via fMRI, rather than the structural exploration of brain anatomy. What both levels of research have in common is that they deal with overlapping, but analytically distinguishable issues of measurement uncertainty, technological limitations, and vagueness which is due to the underlying properties of the brain. Logothetis (2008) investigated the first two issues with regard to the physiological interpretation of the BOLD-signal. He characterized this interpretation of the BOLD-contrast as ambiguous, since different levels of oxygenation alone are insufficient to conclude whether the cortical mircocircuits are largely inhibitory, excitatory or whether inhibition and excitation balance each other out. “Ambiguity” here does not refer to the linguistic notion, where there is an over-determination of conventionally established semantic meanings, but to the missing specificity of the signal when identifying the brain's physiological organization. Since changes in the fMRI signal can include spiking output, neuromodulatory effects, and feedback loops within cortical microcircuits, establishing the convergence of imaging studies with electrophysiological evidence remains challenging (Bartels et al., 2008). But this ambiguity of the signal is not equivalent to its imprecision, although in practice, fMRI can comprise measurement uncertainties due to downstream effects, where activations come from other areas (Tehovnik et al., 2006) or signal blurring across gyri due to the magnetization of brain tissue (Ojemann et al., 1997). What the underspecification of the causal source regarding a signal change has in common with linguistic ambiguity, is that it could be resolved when it is specified what the speaker/signal refers to. Just as the ambiguous term “bank” has two precise and disjunctive meanings (“financial institution” or “edge of a river” cf. Zhang, 1998), the BOLD-signal implies a well-understood physiological entity (inhibitory-excitatory networks) measured by a well-defined unit of measurement (deoxyhemoglobin). Although fMRI alone may not provide the technological means to specify between the different physiological possibilities (Logothetis, 2008), its ambiguity should not be confused with measurement uncertainty or vagueness.

Suppose for a moment one could distinguish between the different physiological interpretations of the BOLD-signal by registering fMRI data to electrophysiological evidence in a hitherto unconsidered way. It is usually assumed by philosophers that vagueness would not be eliminated by such additional information, just like the disambiguation of a term just specifies which one of the precise multiple meanings is expressed, while leaving the fuzzy referential boundary of a vague concept unchanged (Zhang, 1998). This distinction is maintained in the case of measuring brain activity with the fMRI signal, because the source of vagueness here is a more abstract and general property of the brain than its specific physiological organization: The brain is a causally dense system, where almost every neuron is (at least weakly) causally connected to every other neuron (Savoy, 2001). Now the “activation maps” mentioned earlier by Saxe and colleagues, consist of null hypothesis significance tests for each three-dimensional voxel in the brain. The shown activations correspond to a chosen threshold (α-value) which indicates the probability that the data would have been occurred, in case the null hypothesis would have been correct (Gigerenzer, 2004). But since the null hypothesis—that the experimental task had no effect on the data—is for causally dense systems always strictly speaking false, there is no rational justification for setting the significance threshold at any particular value. Moreover, since the statistical parameters between single voxels also change gradually throughout the brain (Huettel et al., 2009), “thresholding at any α inevitably creates artificially sharp barriers between ‘active’ and ‘inactive’ regions” (Klein, 2010, p. 270). In other words, the activity/non-activity distinction is vague because for any significance level there is a vaguely delineated class of *p*-values (such as *p* = 0.049/0.051) for which it is indeterminate whether the sentence “neurons in this voxel are active/are not active” is true or false for a conventionally established but arbitrary threshold (here: α = 0.05). Stipulating such arbitrary thresholds to eliminate the vagueness of “activation” is neither restricted to task-related functional imaging however, but has been also recognized for the construction of probabilistic cytoarchitectonic maps (Amunts et al., 2004) and the study of anatomical and functional connectivity (Passingham et al., 2002). Nor is it a problem is particular to neuroscience, since it applies to any scientific discipline that studies causally dense systems, such as economics (McCloskey and Ziliak, 1996) or ecology (Johnson, 1999), or applies conventional thresholds (most commonly α = 0.05) such as psychology (for an overview see Harlow et al., 1997) or medicine (Sterne and Davey, 2001). But the globality of this problem does not imply that neuroscience cannot tackle it in relationship to the specific implications for the study of the brain, nor does it make impossible that meaningful assumptions about the organization of the nervous system can be made. Rather, it should encourage a discussion about the prospects of quantitative assessments of signal changes in fMRI (Bandettini et al., 2000) and neuropsychological models (Savoy, 2001). Furthermore, considering the issue of vagueness, it is important to note that “significance” is an interest-relative concept (Fara, 2008): what level of *statistical* significance is considered to be *scientifically* significant depends on how many false-positives are accepted for a given research context. “Interest” here means not the individual but the purpose of a whole field of research, relative to which the presence of false-positives is evaluated (e.g., functional localization, cf. Passingham et al., 2002 or neurosurgery cf. Gorgolewski et al., 2011). Taking this into account, the arbitrariness in choosing a particular threshold becomes relativized, as long as significance tests are not used as unquestioned rituals but as check on the experimental data which needs additional reasoning for being validated (Gigerenzer, 2004; Amunts et al., 2004).

Distinguishing vagueness from ambiguity in the case of fMRI studies must not imply that the first one is utterly independent from the technological specifications and methodological assumptions in a specific research context. In fact, it is also recognized in philosophical research that context-sensitivity does include more than just the canonical indexical parameters of time, place, and speaker (Lewis, 1980). More specifically, it has been proposed that for the use of vague predicates, the abilities of the speakers have to be taken into account before evaluating the truth value of a proposition. These abilities are not individual but given through the field of knowledge which determines which propositions are expressible (an approach that has been called “nonindexical contextualism,” cf. MacFarlane, 2007, for vagueness see Åkerman and Greenough, 2010 and Keil, 2010). Such a position can explain the observation of Saxe and colleagues about the laymen's misinterpretation, by pointing to the absent ability of determining which degree of precision is built into a statement which is visualized by a functional activation map. The case of the untrained reader may be an obvious example, but when non-indexical contextualism is combined with the interest-relativity of “significance” described in the last paragraph, it can accommodate for much subtler context-shifts that occur in neuroscientific studies of connectivity, regarding assumptions built into used methodologies (“Anatomical Connectivity and Overlapping Networks” and “Functional Connectivity I: Edge Detection Algorithms and Functional Boundaries”) and the utilization of vague concepts (“Functional Connectivity II: Research Contexts and Combinatory Vagueness”).

## Implications of vagueness for mapping connectivity of the human cerebral cortex

### Anatomical connectivity and overlapping networks

Distinct patterns of axonal connections between different cortical areas represent one criterion of determining anatomical boundaries in non-human and human studies (Felleman and Van Essen, 1991; Passingham et al., 2002). One central assumption is that based on these connections, the brain can be described as consisting of large-scale networks, which are internally segregated into different communities, but nevertheless allow for high integration through internodal links (Tononi et al., 1994). This feature of networks of any kind is called “small-worldness” and can be represented by graph-theoretical analysis, where nodes are maximally clustered (indicating high segregation) and the characteristic path length between any two nodes is not significantly different from randomly distributed networks (indicating high integration, Watts and Strogatz, 1998). While small-worldness could be demonstrated for functional connectivity in the macaque cortex, because anatomical connections could be directly traced *in vivo* (Stephan et al., 2000), determining these connections for the human cerebral cortex remains challenging because they can only be indirectly measured non-invasively. Moreover, the question remains whether brain networks share with other real networks the property of an overlapping community structure, where nodes can belong to several communities (Palla et al., 2005).

In order to tackle the last question, Wu et al. (2011) constructed a structural brain network based on the measurement of regional gray matter volume changes detectable in MRI scans. Based on the assumption that changes in the morphometry correlate with different anatomical connections between cortical areas (Mechelli et al., 2005), Wu and colleagues parcellated the gray matter images into 90 areas and constructed an interregional correlation matrix between all subjects, which could then be transformed into a binarized and undirected network. They then calculated for which parameter *k* (size of the subgraphs which segregate the network into different communities) the mutual information with the original network was the highest. Since the maximum point was found at *k* = 7, where 15 nodes showed singular or multiple overlap, Wu and colleagues concluded that their analysis revealed “fuzzy boundaries” between communities in the structural brain network.

But can “fuzzy” here be understood in the technical sense of vagueness, i.e., as allowing for borderline cases? It is plausible to interpret the overlapping nodes as the shared boundaries between networks, but that does not imply that this boundary is vaguely defined. In fact, since similar connection patterns in a cluster of areas are also described as a family sharing degrees of resemblance (Passingham et al., 2002), it seems more reasonable to use the notion of “family resemblances” (Wittgenstein, 1953/2001) to interpret overlapping nodes. A family resemblance concept (e.g., “game”) is characterized by having applications that stand in a non-transitive relationship to each other, while not sharing a single constant feature. Soccer and chess are both considered games, but are linked only through an intermittent series of more similar games. The semantic structure of such concepts can be considered to form networks with overlapping attributes (as assumed by prototype theory, cf. Rosch and Mervis, 1975). Thus, if cortical areas could be linked through similar but non-identical connectional patterns, overlapping nodes can be considered as the shared attributes of a family resemblance relation. Although vagueness and family resemblance are not entirely separate linguistic phenomena (see section “Functional Connectivity II: Research Contexts and Combinatory Vagueness”), there are at least two reasons to reject the family resemblance interpretation for this particular study. First, the basis for the structural network of Wu and colleagues were one-dimensional, gradable units of measurement (cortical thickness and gray matter volume), while family resemblant concepts necessarily have multi-dimensional features which can be categorically distinct as well (instead of being gradable). Second, structural networks constructed through morphometric measurements can reveal hierarchical organization (Bassett et al., 2008), while the non-transitivity of family resemblance concepts makes a clustering of members based on cumulative similarity problematic.

If the disanalogy to the notion of family resemblances speaks for interpreting overlapping nodes as cases of vagueness, what could the borderline cases look like and how do they relate to the issues described in the last two sections? The case of setting thresholds arbitrarily at a point where overlap occurs can be ruled out, because the criterion for *k* was maximality of mutual information, and more importantly, the number of overlapping nodes remained constant while applying different cost thresholds (ratio between number of edges and possible edges) to the network. Note that here, Wu and colleagues did not use a threshold that shows the most dissimilar patterns of connections (Passingham et al., 2002), because the purpose of their study was exploratory, rather than testing any hypothesis about functional localization. If these overlapping nodes can be considered predictors of their functions (Palla et al., 2005), however, then the areas could represent borderline cases (in the sense of Passingham and colleagues), in which it is indeterminate whether or not double dissociation studies reveal which functional role a cortical area fulfills.

As the findings of Wu and colleagues were robust over different thresholds and concerned with anatomical rather than functional connectivity, the comparison to vagueness in the cytoarchitectonic study of the brain deserves special attention. The first connection between these two levels is provided by the authors themselves when they write that “the overlapping community structure would be changed by parcellation templates due to different boundaries between brain regions” (13). Here, it seems like the vagueness of cytoarchitectonic (or other) parcellations of the brain gets imported into the abstract representation of network structure, suggesting that the fuzzy community boundaries are analogous to higher-order vagueness, where the vagueness of the object language gets imported into the meta-language (Varzi, 2001). The analogy is not necessarily correct however, since it is possible that the overlapping community structure is an independent organizational principle of the connectivity of the brain, which is preserved even if cytoarchitectonic parcellation schemes could be made perfectly precise. Moreover, since the distinction between anatomical and functional connectivity is itself blurry for the development of new axonal connections (cf. Fingelkurts et al., 2005), the microscopic study of structural cell properties should not be the only modality that evaluates the vagueness of cortical networks. Despite these restrictions, is there a sense in which overlapping nodes are related to the gradual transitions observed in cytoarchitectonic studies? Such a relation is in principle possible, since it could be shown in rhesus monkey studies that the anatomical connectivity patterns can be predicted from the laminar structure of different cortical layers (Barbas and Rempel-Clower, 1997). Whether this prediction does hold in the case of human anatomical connectivity as well cannot currently be determined empirically, because regional gray matter volume or densities measured in 1 mm^3^ MRI voxels are not equivalent to cytoarchitectonic cell packing densities (Mechelli et al., 2005). But this impossibility is not only a problem of resolution but also a conceptual one: “gray matter” is a not a sortal concept that picks out individual entities in the world (for an overview see Pelletier, 2010). Thus, the question “how much gray matter is in a cortical area?” can only be answered by using the appropriate unit of measurement (e.g., mass per unit of volume). Consequently, in order to transform gray matter volume data into a structural network, a parcellation of cortical areas that utilizes sortal concepts (such as “neuron” or “axon”) has to be already in place (no matter how vaguely the cortex is parcellated by them). The same caution holds for comparing the determined overlapping community structure to diffusion tensor imaging (DTI), where axonal connections are estimated from MRI scans by calculating the least hindered diffusion of water throughout the each voxel. Since DTI methods are currently unable to detect the exact origination and termination points of axonal connections, there are no clear cases of precisely segregated connections (characterized as “ground truth” by Jbabdi and Johansen-Berg, 2011) which could serve as a comparison class to the alleged borderline case of overlapping connectivity of subnetworks (i.e., different networks sharing anatomical axonal connections). In other words, given the current technological restrictions, the discovered fuzzy boundaries between communities in the structural brain network cannot be regarded as a case of extensional vagueness yet, because the relationship to the underlying anatomical features remains to be determined. But this technological shortcoming should again not be taken to rule out that this study provides evidence for the intensional vagueness of structurally connected brain networks.

### Functional connectivity I: edge detection algorithms and functional boundaries

The previous section already showed, to some extent, how assumptions about the organizational properties of the cerebral cortex are built into the algorithms used to computationally analyze brain connectivity (Rubinov and Sporns, 2010). In this section, the implications of vagueness will be further explored for algorithmic procedures which are used to define functional boundaries via resting state fMRI data. Unlike task-related fMRI, resting state studies correlate time series of slow, low-frequency hemodynamic responses across different areas, without testing for experimentally induced differential changes in brain activation. Thus, the acquired BOLD-data reflect the statistical history of co-activated areas that are functionally connected to each other. The objective of this method is to delineate functional boundaries in the cortex which are not assessable on the basis of cytoarchitectonic studies at the microscopic scale or task-based functional localization alone (although these may be ultimately connected, cf. Stephan et al., 2000; Passingham et al., 2002).

In a recent study, Cohen et al. (2008) proposed a method to reveal the putative functional boundaries of the human cortex by using the Canny edge detection algorithm (Canny, 1986) on resting state fMRI data. To apply the algorithm, the simultaneously acquired structural MRI images were used to generate a two-dimensional Cartesian grid by flattening the cortical surface with as minimal distortion as possible. The Cartesian grid could then be used to define volumetric seed regions by which the pre-processed resting state functional connectivity MRI data could be localized in the cortex. By correlating the time course of one seed with the time courses of every other voxel, volumetric correlation maps where created to compare the similarity of the connectivity profiles of each seed region (expressed by the coefficient eta^2^). To detect the putative functional boundaries between areas, the eta^2^ coefficients (values between 0 and 1 indicating no similarity or complete identity) were thresholded at two points:

To prevent hysteresis, if the magnitude of the pixel is below the low threshold, it is set to zero, while if the magnitude is above the high threshold, it is considered an edge. If the magnitude of the pixel is between the two thresholds, then the location is only considered an edge if there is a neighboring pixel that itself has a gradient above the high threshold

(Cohen et al., 2008, p. 49).

In using that method, Cohen and colleagues are assuring to “identify and differentiate locations with strong, spatially coherent peaks as being different from locations that are relatively smooth or have incoherent gradient peaks” (Cohen et al., 2008). The aim of eliminating the apparent uncertainty whether or not values in between the two thresholds should be regarded as an edge, is analogous to the aim of the philosophical theory of supervaluationism, which was developed in order to preserve the principles of classical logic from problems created by the Sorites-paradox (Fine, 1975). In particular, borderline cases seem to refute the principle of bivalence (i.e., that propositions are either true or false) because they have truth value gaps (see section “The Philosophical Concept of Vagueness”). Supervaluationism attempts to preserve the bivalence principle by considering only those cases as borderline cases which are true under some, but false under other precisifications, while the clear cases are the ones which are true and the clear non-cases are the ones being false under all admissible precisifications (i.e., being super-true or super-false). The advantage of the theory is that after the process of supervaluation, all the borderline cases have a determinate truth value under some precisification and thus do not possess truth value gaps. Equivalently, the utilization of the binary edge detection algorithm by Cohen and colleagues assigns to eta^2^ coefficients the property of being an edge under some precisifications (i.e., if eta^2^ of the neighboring pixel is above the threshold) and not under others (if neighboring eta^2^ is between or below thresholds). If the analogy between these two approaches is correct, then determining functional boundaries via the binary edge detection algorithms suffers from higher-order vagueness just as supervaluationism does. Because the boundary between clear cases, borderline cases, and non-cases is fuzzy, the term “admissible precisification” in the supervaluationist semantic is itself vague (cf. Williamson, 1994; Varzi, 2001). In the case of the binary edge detection, however, higher-order vagueness is imported through the use of arbitrary thresholds (see section “Thresholds and Measurement of Neural Activity with fMRI”), which serve the purpose to ensure spatial stability across short stretches of the eta^2^ profiles (Cohen et al., 2008), because what counts as “short” is vague. This observation does not discredit the algorithm as an inappropriate method for defining boundaries *per se*, but it points out that its methodological assumptions may be inappropriate to deal with every part of the cortex equally. Just as some parts of the brain were more prone to vagueness using cytoarchitectonic criteria due to gradual transitions, some parts of the brain may have more gradually changing functional connectivity profiles than others. The study of Cohen and colleagues confirms this assumption, since they report three seed regions for which the connectivity profile between the angular and supramarginal gyrus did not show an abrupt change.

Analyzing methods in isolation, however, would give the false impression of how neuroscientific research is in practice organized, since explaining neural phenomena requires interrogations from different disciplinary perspectives and at multiple scales (or levels, cf. Craver, 2007). Such a characterization can be made about the example discussed above, to the extent that in a different study, Nelson et al. (2010) used the binary edge detection algorithm together with other methods (task-related fMRI, network analysis) to assemble convergent evidence about the functional parcellation of the human left lateral parietal cortex. Although philosophers usually acknowledge the importance of convergent evidence in neuroscientific explanations (Churchland and Sejnowski, 2000), it is not by itself clear whether convergence points toward a fundamental level of explanation (Craver, 2007). It also remains to be determined by further analysis how the notion of convergent evidence relates to the contested view of convergent realism in philosophy of science (for critiques see Laudan, 1981; Hoyningen-Huene, 2011). What is important for the present purpose, however, is that vagueness can transverse these different research strategies as well. Because the binary edge detection algorithms alone cannot be interpreted as reflecting the underlying different functional connectivity profiles, Nelson and colleagues also constructed a network for the left lateral parietal cortex, based on defining neighborhood seed regions across the cortex which had correlated connectivity profiles with the ROIs. They were able to subdivide this network into entirely distinct communities, following the purpose of functional localization, where potentially overlapping (i.e., intensionally vague) neighbors are excluded in the presence of abruptly changing connectivity profiles.

But it becomes evident from the section “Anatomical Connectivity and Overlapping Networks” and the reported intermediate seed regions from Cohen and colleagues that this community structure is not the only possible or the most appropriate parcellation of a cortical area. A study by Yeo et al. (2011) provides another recent example where one possible way to deal with connectivity profiles is expressed. These authors studied intrinsic functional connectivity of the whole cortex on a population basis. One of the findings was a gradual transition in the functional correlations between areas V1 and V3, when shifting from input from the central to the peripheral visual field. While expressing the uncertainty how to accommodate for this result, an uncertainty which is common when dealing with borderline cases, Yeo and colleagues also conclude in their discussion that this gradual transition can be turned into an “abrupt division into central and peripheral networks when assignments into distinct networks are forced, providing a convenient way to map the topographical organization across visual areas” (1151). But finding a convenient way to map gradual transitions is not sufficient to deal *reasonably* with vagueness, because of the interest-relativeness of significance and nonindexical contextual factors. These parameters become especially important when parcellation schemes travel outside the basic research into practical settings, where relying on boundary locations has real-world consequences (e.g., clinical applications such as neurosurgery). Here, mistakes could be made when the ability to discriminate different degree of precision is not taken into account (e.g., working with invasive instruments such as scalpels, instead of imaging). Thus, neuroscientists need to be aware that arbitrarily chosen boundaries do not simply correspond to the brain's underlying structure in a one-to-one manner (just as functional boundaries and cytoarchitectonic do not need to, cf. Huettel et al., 2009). How much vagueness is allowed into the scientific descriptions of nature depends both on the purposes of the researcher and on how adequately a concept is supposed to capture the underlying properties of the object in question.

### Functional connectivity II: research contexts and combinatory vagueness

While the last section explored from what level the vagueness of delineating functional boundaries gets imported into resting state connectivity studies, this section asks whether the character of the studied neural phenomenon itself has implications for the concept of “functional connectivity.” In order to address this question it will be more helpful to begin in a reverse manner by looking at the conceptual side first, since the term “functional connectivity” has been criticized by Horwitz (2003) as an elusive concept. The reason for this critique is the observation that researchers allegedly investigate the same phenomenon while actually conducting experiments which determine functional connectivity based on considerably different features: the use of various imaging modalities (EEG, PET, MEG, fMRI) results in different spatial and temporal resolutions as well as to whether connectivity is assumed between neural activity, neuronal ensembles or large-scale interactions. Furthermore, there are multiple ways in which experimental parameters can be correlated (across or within conditions, subjects, or time courses). Now although it is correct to say that due to these different possibilities, there can be unclarity about whether the same aspect of cortical organization is explicated by various experiments, it is misleading to assign the rather pejorative term of elusiveness to “functional connectivity” because of that fact. Such a characterization is quite similar to the assumption that vagueness is a deficit of natural language which has to be eliminated by formalization, a position expressed by some philosophers of logic (e.g., Frege, 1893/1903; Russell, 1923). But after the focus on the language use of ordinary speakers revealed that vagueness can play a positive role in communication (Austin, 1975; Wittgenstein, 1953/2001), the deficit interpretation is not upheld anymore in the contemporary theoretical literature (Williamson, 1994; Hyde, 2007). Furthermore, there is no reason to consider the concept as ill-defined since—as Horwitz also acknowledges—the neuroscientific community has reached consensus by using the definition of Friston (1994): “functional connectivity” means “temporal correlation between spatially remote neurophysiological events.” The definition provides an example of two earlier observations concerning vagueness in neuroanatomy. First, it shows that vagueness is not necessarily eliminated by reaching consensus (as proposed by Saxe and colleagues), since “spatial remoteness” is a vague concept which allows for different borderline cases depending on the resolution of the imaging modality (how small can “spatially remote” be?). Second, vagueness is overlapping with other issues in empirical research (“Thresholds and Measurement of Neural Activity with fMRI”) since “neurophysiological event” is an ambiguous term which can refer to different acting entities (e.g., single firing neurons, differentially active areas). While spatial remoteness is a case of one-dimensional degree-vagueness, the presence of one of these events is a necessary (albeit alone insufficient) condition in order to count a temporal correlation as functionally connected.

Taking these facts together, the characterization of functional connectivity should not be considered as an elusive but as a combinatorially vague concept (Alston, 1967). Combinatorially vague concepts (e.g., “religion”) are a subclass of family resemblance concepts (see section “Anatomical Connectivity and Overlapping Networks”) which allow for borderline cases of application (e.g., “religion” to the Quaker movement or ideologies such as communism). Although there are clear examples (e.g., Catholicism), the list of features characterizing such a concept does neither comprise a list of necessary and together sufficient conditions, nor does it require the shared characteristics of the “family members” to stand in the same logical relation to each other. If the different experimental possibilities pointed out above are now interpreted as the feature list for the concept of functional connectivity, then this concept admits for borderline cases of application because it is not only unclear how spatially remote two events have to be (degree-vagueness), but also because different family resemblant events can fulfill the definition. However, there are two reasons why these borderline cases cannot be easily detected. First, every imaging modality and analysis step has certain advantages over others, but no single one is superior in every respect, thus making the existence of a “perfect instance” (where all features of the list are fulfilled) of functional connectivity unlikely. Second, the status of a clear case does not require a cumulative fulfillment of the feature list, where the ones with more features are always “clearer” (Wittgenstein, 1953/2001). That borderline cases only become visible when different research contexts are compared, can be elucidated by the response of Fingelkurts et al. (2005) to the critique of Horwitz (2003). These authors proposed a neurophysiological interpretation of functional connectivity by using a combined EEG/MEG study to measure phase synchronization of electrical signals at both local and global spatial distances. When this research context is taken as a reference frame for comparing other functional connectivity studies, borderline cases arise due to lack of certain features (e.g., high temporal specificity for an fMRI study) or the missing comparability of experimental procedures (e.g., within vs. across correlations).

How can researchers possibly deal with this type of vagueness? Note first that the quantification of the signal (compare “Thresholds and Measurement of Neural Activity with fMRI” section) is of no help here because dealing with combinatory vagueness requires to decide whether or not a certain feature should count as a condition for application of a concept (although weighing these criteria may require some ordinal scale). Thus, even if a quantitatively interpretable threshold for spatial remoteness is available and the research context is held fixed, functional connectivity remains combinatorially vague due to the ambiguity of “neurophysiological event.” At this point of the reconstruction, the consensual solution seems the most plausible and straightforward one: once disambiguated, researchers find that the term “functional connectivity” has several perfectly precise and distinct meanings which can be now labeled anew. But this solution loses its plausibility once the object side is considered instead of the conceptual side of the problem. Although the linguistic phenomena of combinatory vagueness and ambiguity both share the feature of polysemy (having multiple meanings), they are not identical. Because in the former case, all the meanings share a common core of extensions (Pinkal, 1995), while in the latter, extensions do not usually overlap (recall the “bank” example in “Thresholds and Measurement of Neural Activity with fMRI”). The disambiguation of the neurophysiological interpretations does therefore not reveal entirely different neuroscientific phenomena, but rather different aspects of the same organizational principle of the brain. Therefore, the question neuroscientific researchers should ask is whether functional connectivity is a principle general enough that it can be manifest at different levels of neuroscientific research, even in the absence of a clear correspondence to the anatomical connections. The practical relevance of combinatory vagueness can be exemplified by considering once more the vagueness of networks formed by functionally connected areas: Networks are not only gradually vague with respect to the resemblance degree of connectivity profiles (“Anatomical Connectivity and Overlapping Networks”), but also combinatorially vague with respect to the number of communities. Individuating single communities follows a feature list which does not have to be fulfilled in every research context, i.e., having an anatomical counterpart, sharing topographical features with this counterpart etc. Addressing these questions may imply that convergence of evidence (“Functional Connectivity I: Edge Detection Algorithms and Functional Boundaries”) from different subfields in the neuroanatomical community does not *per se* lead to increasing precision, but could reveal different types of vagueness as well (following Alston's criterion that the definiens should match the definiendum in its vagueness). If that observation is correct for the case discussed here, then the disambiguation of “functional connectivity” would not lead to better research because it would misconstrue an important characteristic of how cortical organization is to be understood.

## Conclusion and outlook

This paper provided a preliminary conceptual framework for understanding vagueness in neuroanatomy at different levels of research. It should encourage empirical researchers to engage in a discussion about whether the concepts used in scientific practice express a notion of anatomical and functional organization which is appropriate to understand the complex system of the human brain. The importance of such a discussion is supported by a recent trend in philosophy of science, which emphasizes the interdependence of concept formation and experimentation in empirical science (Wilson, 2006; Rouse, 2011). Note that the philosophical discussion of vagueness is in an important sense similar to the neuroscientific investigation of functional connectivity: both are best seen as enterprises which are open toward the future, and some of the positions presented here are the outcome of an intensive theoretical discussion of the last 40 years of philosophy of language and logic. A characteristic difference of the approach proposed here, however, is that particular fields of knowledge like neuroscience can be used to assess how adequately general theories of the phenomenon of vagueness can deal with particular issues of indeterminacy and fuzziness.

With this characterization in place, the results of the above analysis can be summarized as follows: “Gradual Transitions in Cytoarchitectonic Studies of Brain Anatomy” revealed that the basis of vagueness in neuroanatomy is one-dimensional gradual transitions of structural anatomical properties of the cortex. To deal with this type of vagueness, researchers characterize cortical regions in multiple modalities (cyto-, myelo-, and chemoarchticture), but in some cases all of these modalities can reveal a transitional zone (such as 9/46). “Thresholds and Measurement of Neural Activity with fMRI” proceeded to discuss the issue of statistical thresholding in causally dense systems like the brain. Here, quantification of the fMRI signal and neuropsychological models may narrow the degree of arbitrariness, but that does not provide a general solution to vagueness because significance is an interest-relative concept and therefore dependent on specific neuroscientific research contexts. “Anatomical Connectivity and Overlapping Networks” and “Functional Connectivity I: Edge Detection Algorithms and Functional Boundaries” discussed then how these two types of vagueness get imported into the study of connectivity in the human cortex. The overlapping community structure of structural brain networks suggests an intensional vagueness of delineating cortical areas based on anatomical connectivity, because the indirect measure of axonal connections does not provide evidence for actual borderline cases, but only possible ones. In case of determining functional boundaries, the analysis of edge detection algorithms showed that the decision rule for classifying voxels in non-, borderline, and clear cases of edges suffers from higher-order vagueness in the same way supervaluationism does. This observation is supported empirically by gradual transitions in functional connectivity profiles. “Functional Connectivity II: Research Contexts and Combinatory Vagueness” then asked more generally, whether the neural phenomenon of functional connectivity is a source for the combinatory vagueness of comparing its manifestation in different experimental settings. If that type of vagueness indicates an organizational principle of the brain, then a discussion about the conceptual articulation of properties of the human cortex is needed.

Despite the theoretical discussion in this paper, there are also further possibilities to deal with vagueness in empirical studies. To characterize indeterminacy, some structural MRI researchers are using fuzzy clustering methods to describe the volumetric and cardiac features of resonance images (Kobashi et al., 2007; Kurkure et al., 2009; Lin et al., 2010). Fuzzy logic applies different degrees of truth to the propositions expressed by sentences that contain vague predicates (Zadeh, 1965). Functional neuroanatomists could pursue a fusion of this “fuzzy mode of medical thinking” (Seising, 2006) and the application of fuzzy set theory already in place in neural networks (Kosko, 1992) by integrating these clustering methods in the study of anatomical and functional connectivity. It should be also noted, however, that it has been repeatedly questioned by philosophers whether the fuzzy logic approach provides a satisfying solution to vagueness, because it is over-precisifying statements whose communicative use is generated by their fuzzy referential boundaries (cf. Keil, 2010). More particularly for vagueness in neuroanatomy, quantification does not provide a solution to combinatory vagueness (see section “Functional Connectivity II: Research Contexts and Combinatory Vagueness”). Thus, using philosophical vocabulary to analyze issues in the empirical study of the brain can produce a fruitful discourse beside the more traditional encounters between brain scientists and philosophers regarding the relationship between mind and body.

### Conflict of interest statement

The authors declare that the research was conducted in the absence of any commercial or financial relationships that could be construed as a potential conflict of interest.

## Footnotes

^1^The perspective of this paper is to understand neuroanatomical research from a philosophical viewpoint. The presented neuroscientific examples and the arguments based on them are not meant to be an external criticism of these studies, but are used to make a general, constructively critical point about issues of vagueness in the study of the human cortex. Any empirical questions generated by this analysis have to be left open for further neuroscientific research.

^2^Transitional zones are also found in non-human animals, such as area 17/18 in the cat visual cortex (Payne, 1990), which is not only cytoarchitectonically defined as intermediary between area 17 and 18, but also represents a grey zone with regard to neuronal preference of spatial frequencies (Ohki et al., 2000). Since the focus of this special issue is the neuroanatomy of the human cerebral cortex, area 9/46 will be discussed in greater detail. It should be noted that the history of anatomical research on this area is considerably more complex than presented here. Not only did Walker (1940) classify parts of area 9 as area 46 not present in earlier cortical maps of the macaque brain (Brodmann, 1905; Vogt and Vogt, 1925), but also did these delineations not resemble the designations of area 9 and 46 for the human brain (Brodmann, 1909). In order to accommodate for the research needs of both communities, Petrides and Pandya (1999) created a uniform vocabulary for human and primate anatomy, and furthermore subdivided area 9/46 in a dorsal and ventral portion based on comparative studies. Note however, that this conventional definition of uniform vocabulary does not change the transitional nature of area 9/46 as outlined by Rajkowska and Goldman-Rakic (1995a).

^3^If the cytoarchitectonic properties are interpreted as identity conditions for cortical areas, then these areas could be even considered vague objects (Tye, 1990), although this position remains controversial and has been criticized repeatedly (Russell, 1923; Varzi, 2001).
